# Measles Virus: Identification in the M Protein Primary Sequence of a Potential Molecular Marker for Subacute Sclerosing Panencephalitis

**DOI:** 10.1155/2015/769837

**Published:** 2015-10-26

**Authors:** Hasan Kweder, Michelle Ainouze, Joanna Brunel, Denis Gerlier, Evelyne Manet, Robin Buckland

**Affiliations:** ^1^CIRI, International Center for Infectiology Research, Université de Lyon, 69007 Lyon, France; ^2^Inserm, U1111, 69007 Lyon, France; ^3^Ecole Normale Supérieure de Lyon, 69007 Lyon, France; ^4^Centre International de Recherche en Infectiologie, Université Lyon 1, 69007 Lyon, France; ^5^CNRS, UMR 5308, Lyon, France

## Abstract

Subacute Sclerosing Panencephalitis (SSPE), a rare lethal disease of children and young adults due to persistence of measles virus (MeV) in the brain, is caused by wild type (wt) MeV. Why MeV vaccine strains never cause SSPE is completely unknown. Hypothesizing that this phenotypic difference could potentially be represented by a molecular marker, we compared glycoprotein and matrix (M) genes from SSPE cases with those from the Moraten vaccine strain, searching for differential structural motifs. We observed that all known SSPE viruses have residues P64, E89, and A209 (PEA) in their M proteins whereas the equivalent residues for vaccine strains are either S64, K89, and T209 (SKT) as in Moraten or PKT. Through the construction of MeV recombinants, we have obtained evidence that the wt MeV-M protein PEA motif, in particular A209, is linked to increased viral spread. Importantly, for the 10 wt genotypes (of 23) that have had their M proteins sequenced, 9 have the PEA motif, the exception being B3, which has PET. Interestingly, cases of SSPE caused by genotype B3 have yet to be reported. In conclusion, our results strongly suggest that the PEA motif is a molecular marker for wt MeV at risk to cause SSPE.

## 1. Introduction

Subacute Sclerosing Panencephalitis (SSPE) is caused by measles virus (MeV), a member of the genus* Morbillivirus* in the family* Paramyxoviridae*. The enveloped MeV virion contains a nonsegmented negative-strand RNA genome encoding six structural proteins: N, P, M, F, H, and L. The glycoproteins H (hemagglutinin) and F (fusion) project from the virion membrane as spikes. The H protein is responsible for attachment to the cellular receptors and the F protein for the consequent fusion of the virion membrane with the host cell's plasma membrane [1]. In the infected cell, the glycoproteins accumulate in the plasma membrane where they interact with cellular receptors on neighboring uninfected cells to cause cell-cell fusion (syncytia formation). The matrix protein M is believed to line the inner surface of the plasma membrane of the infected cell, interacting with the cytoplasmic tails of the glycoproteins [2, 3]. As far as cellular receptors for MeV are concerned, the wt strains use SLAM (CD150) whereas the vaccine and laboratory strains use both SLAM and CD46 [4]. Although CD46 is ubiquitously expressed in the human body, the expression of SLAM is restricted to cells of the immune system [4]. A long searched for third receptor, nectin-4, has been recently identified [5, 6] which allows MeV infection via epithelial cells. Intriguingly, of the three MeV receptors, only CD46 is expressed in the CNS [7].

SSPE is a rare fatal disease of children and young adults that is due to a persistent MeV infection of the brain. SSPE symptoms appear several years after an apparently banal wt MeV infection; SSPE cases caused by vaccine strains of MeV have never been reported. Although more than 80 years has passed since the disease was described by Dawson [8], no mechanisms responsible for the pathogenesis of SSPE have been identified (for a review see [9–12]). However, MeVs from SSPE neurological tissue are characterized by multiple mutations in the H, F, and M proteins of the viral envelope that impairs their function [13–16]. Measles inclusion body encephalitis (MIBE) also infects the human brain but is caused by both wt and vaccine strains of MV [17, 18]. Moreover, in contrast to SSPE, MIBE only occurs in immunocompromised individuals, such as AIDS patients, and presents within weeks of infection rather than years.

In a previous study, we showed that vaccine/laboratory strains of MeV are susceptible, like wt MeV strains, to accumulate mutations in their H, F, and M proteins under immune pressure [19]. However, it would appear that, unlike wt MeV, they lack a phenotypic marker that allows spread and persistence in the CNS.

The working hypothesis for the present study was thus that the capacity to spread and persist in the human brain is somehow intrinsic to wt MeV. Potentially this could be due to sequence differences with laboratory/vaccine MeV strains. We therefore decided to compare the primary sequences of SSPE MeV genomes with those from vaccine strains searching for a molecular marker common to all SSPE MeV strains. Initially, we compared primary sequences encoding the H protein but as our previous study [19] had indicated that mutations in both the F protein and M protein can affect the 3D conformation of the H we extended our search to the primary sequences encoding these latter proteins. Comparing SSPE MeV genomes with that from the Moraten vaccine strain of MeV we observed a triresidue motif PEA (P64, E89, and A209) that is always present in the M proteins from SSPE cases but which is, respectively, SKT (S64, K89, and T209) in the Moraten vaccine M protein. Moreover, these residue identities are the same for all vaccine/laboratory strains except that in some residue 64 is proline (hence PKT).

We hypothesize that to cause SSPE a wt MeV strain (or wt MeV genotype for that matter) should have the PEA motif in its M protein. By consulting published sequences available for the 23 wt MeV, we found that only 10 of these include the sequence for the M gene (obtained by direct RT-PCR amplification from brain tissues [20]). Interestingly, all have the PEA motif except genotype B3, which is PET. This intrigued us because B3 is the most prevalent genotype in Sub-Saharan Africa [21], where, despite the hyperendemicity of MeV, the reported prevalence of SSPE cases is unexpectedly low [22]. Even though it is possible that there has been an underreporting of SSPE cases in this region, B3 cases have occurred elsewhere in the world, including the USA. Importantly for our hypothesis, despite an extensive search in the literature, we were unable to find a single SSPE case involving this genotype.

Serendipitously, two B3 genotype strains are available in our laboratory, Lys-1 [23] and G954 [24], so that we were able to compare their capacities for cell-cell fusion and virus production, with a PEA motif-containing D4 genotype virus, Lys05/06. The results suggested that B3 genotype strains produce less virus than PEA motif-containing genotype strains, but to confirm that this was due to the nature of the M protein tri-residue motif, we turned to reverse genetics.

MeV recombinants were thus constructed in which elements of the wt PEA motif were introduced into the vaccine strain's SKT motif within the gene encoding the M protein. By comparing the phenotypes of these different recombinants with regard to their capacities for cell-cell fusion and virus assembly, we obtained results that strongly support the hypothesis that the M protein triresidue motif PEA is important for the spread of wt MV and hence SSPE pathogenesis.

## 2. Materials and Methods

### 2.1. Cells

Vero cells and vero/hSLAM (vero cells constitutively expressing human SLAM) were maintained in Dulbecco's modified Eagle's medium (DMEM) supplemented with 10% fetal bovine serum (FBS), 2 mM L-glutamine, 100 U/mL penicillin, 0.1 mg/mL streptomycin, and 10 mM HEPES. CHO/hSLAM (Chinese hamster ovary cells constitutively expressing human SLAM) were maintained in F12 medium containing 10% fetal bovine serum (FBS), 100 U/mL penicillin, 0.1 mg/mL streptomycin, and 1X MEN nonessential amino acids. The human “helper” cell line 293-3-46 stably expressing the N and P proteins and T7 RNA polymerase were maintained in DMEM medium with 10% FBS, 2 mM L-glutamine, 1.2 mg/mL G418, and 10 mM HEPES.

### 2.2. Viruses

Three wt MeVs were used in this study: Lys-1 and G954 (both B3 genotype), Lys05/06 (D4 genotype). Eight recombinants were built and rescued using a Moraten vaccine strain reverse genetics system (a kind gift from Roberto Cattaneo).

### 2.3. Production of Moraten M Gene Mutants

Mutations in residues 64, 86, 89, and 209 of the M gene of Moraten strain of MeV were introduced separately or in combination using QuickChange kit (Stratagene) according to the manufacturer's instructions. These mutations were introduced into the gene encoding MeV-M cloned into a shuttle plasmid p588 containing the N, P, M, and F genes of Moraten MeV. Then the M gene of this plasmid was cloned, using the In-Fusion HD cloning kit (Clontech), into another plasmid, p698, which contained the totality of the Moraten genome except for the deleted M gene. This plasmid was used in the production of recombinant viruses. All mutations were verified by DNA sequencing.

### 2.4. Production of Moraten Recombinants

293-3-46 cells cultured overnight in 6-well tissue culture plates were transfected with 10 *μ*g of p698 containing the mutated M gene, together with 40 ng of the plasmid pEMC-La, which encodes the MeV polymerase L protein, using the Promega transfection kit (Mammalian Transfection System, calcium phosphate). 16 h after transfection, the medium was replaced with antibody-free medium. 1 h later, 293-3-46 cells were subjected to thermal shock at 42°C for 3 h. After 48 h, cells were gently detached by squidging using the medium and added to 100 mm culture vessel containing vero/hSLAM cells. After 2 to 3 days, syncytia in overlaid vero/hSLAM cells were individually picked and transferred to vero/hSLAM cell monolayers in 75 cm^2^ flasks. Finally, we obtained 6 recombinant viruses named according to the amino acids occupying the triresidue motif (aa 64, 89, and 209) of the M protein: SKT (S64, K89, and T209); PKT (P64, K89, and T209); SET (S64, E89, and T209); SKA (S64, K89, and A209); PET (P64, E89, and T209); and PEA (P64, E89, and A209). Two additional recombinants were built and rescued: S(R)KT (S64, R86, K89, and T209) and P(R)ET (P64, R86, E89, and T209). It should be noted that residue 86 is K in the recombinant viruses SKT, PKT, SET, SKA, PET, and PEA. The M gene of all recombinants was sequenced to confirm the mutagenesis.

### 2.5. Virus Amplification and Titration

A virus stock was made following a second passage of amplification: cells with 2 mL of medium were frozen at −80°C overnight 2-3 days after infection when the majority of cells showed fusion/syncytium formation. Then the medium was thawed and harvested and the virus stock titrated. Cells in 96-well tissue culture plates were inoculated with 1/10 serially diluted culture medium samples for 1 h at 37°C. Then, the inocula were removed and new medium was added to each well. After 4 days, the number of infected wells was counted and the 50% tissue culture infective dose (TCID_50_) and the plaque-forming unit (PFU) were calculated.

### 2.6. SLAM- and CD46-Dependent Fusion Assay

Each virus was studied to determine its capacity to induce the fusion in the presence of either SLAM or CD46 as cellular receptor by using CHO/hSLAM cells or vero cells (CD46+), respectively. The cells in 6-well plates were infected at a m.o.i of 0.01. Cell-cell fusion in infected cells was quantified as described previously [25]. Briefly, 30–36 h after infection, images of ten microscope fields were taken randomly and the proportion of nuclei in syncytia relative to the total number of nuclei was determined by counting.

### 2.7. Cell-Free Virus and Cell-Associated Virus Titrations

Vero cells or CHO/hSLAM cells were infected at a m.o.i of 0.1 of recombinant virus. 48 h after infection, the culture media were harvested, centrifuged at 3000 rpm for 5 min at 4°C, and stored at −80°C until being used for further analysis to determine cells-free virus. In addition, infected cells were also frozen at −80°C overnight. They were then thawed and harvested and the supernatant used to determine the level of cell-associated virus. Thereafter, the titration of cell-free virus and cell-associated virus was made as described above.

### 2.8. Confocal Microscope Study for the Localization of MeV Proteins, H, F, and M

Vero SLAM cells grown on glass cover slides in 12-well culture plate were infected with recombinant viruses at 37°C for 1 h. Then the medium was changed with medium containing the anti-MeV fusion tripeptide FIP [26]. Cells were subjected to immunofluorescence 24 h after infection. Three antibodies were used, anti-H mAb BH129, anti-F mAb Y503, and anti-M mAb8910 (Millipore). The antibodies were labelled using Zenon Mouse IgG Labeling Kits (Molecular Probes). Anti-H mAb BH129 was stained with Alexa Fluor 488, anti-F mAb Y503 with Alexa Fluor 555, and anti-M mAb8910 with Alexa Fluor 647. Cells were first washed with PBS 1x. Then the live cells were incubated only with labelled anti-H and anti-F for 1 h at 4°C. Next, cells were washed with PBS, fixed with 3% PFA, and permeabilized with 0.1% Triton X-100 for 10 minutes at RT. Subsequently, cells were washed with blocking solution (0.2% Tween 20, 2% BSA, and 5% glycerol in PBS) and incubated in blocking solution for 10 minutes. Cells were first incubated with labelled anti-M for 1 h at 4°C and then the slides were prepared for confocal microscope study. Laser argon, laser 561, and laser 633 were used for H, F, and M, respectively. The specimens were studied in two steps, H and M in one step and F in another step to avoid interference between the emission signals of H, F, and M.

## 3. Results

### 3.1. Identification of a Potential Molecular Maker for SSPE in the wt MeV-M Protein

**Figure 1:**
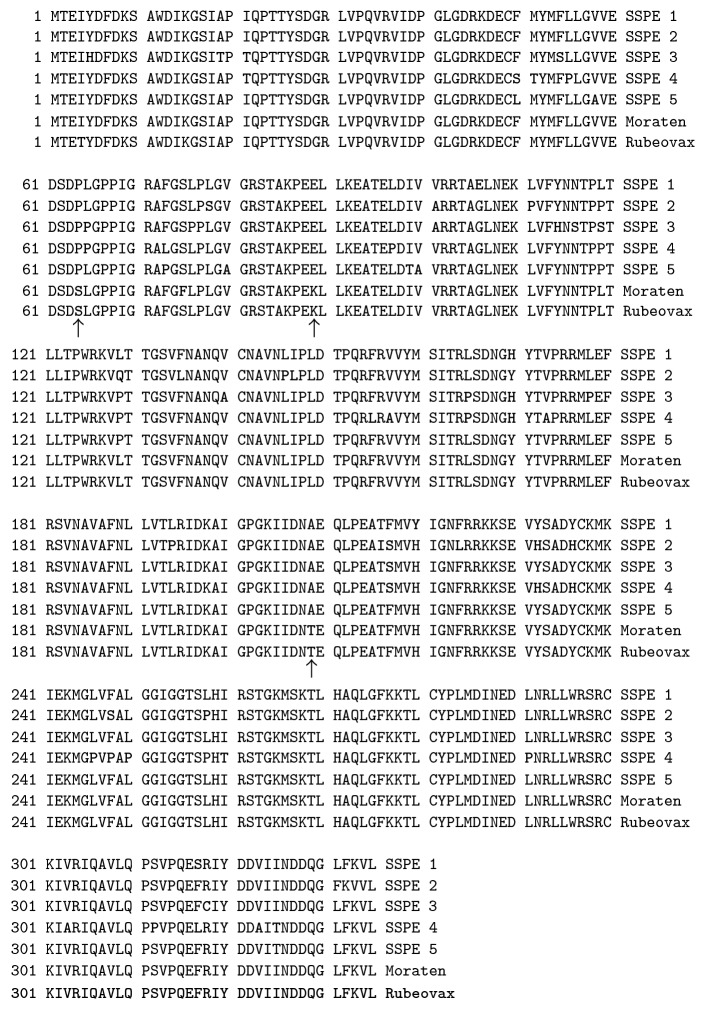
Primary sequence comparison of M protein genes from five SSPE cases [20] and two MeV vaccine strains. Accession numbers SSPE1 (London) AF503528; SSPE2 (Nottingham) AF503530; SSPE3 (Cardiff) AF503531; SSPE4 (Belfast87) AF503526; SSPE5 (Belfast88) AF503524; Moraten (vaccine) AF266287; Rubeovax (vaccine) AF266289.

This study's starting point was the observation that SSPE is caused only by wt MeV, never by MeV vaccines [27]. This suggests that wt MeV strains possess a phenotypic marker that vaccine strains lack. Hypothesizing that such a phenotypic marker could be represented by an associated molecular marker we decided to compare H, F, and M sequences from SSPE cases with those from vaccine strains searching for differential structural motifs. We were unable to identify any type of molecular marker that differentiated SSPE glycoproteins from their vaccine counterparts except for the differences at residues 481 and 546 in the H protein that have been shown to play a role in allowing vaccine strains to use CD46 as a receptor in addition to SLAM [28, 29]. However, comparison of five SSPE case M protein primary sequences with the Moraten and Rubeovax vaccine M proteins [30] revealed the presence of a triresidue motif at residues 64, 89, and 209 that appears to differentiate SSPE and vaccine M proteins (Figure 1). All five SSPE cases have the residues proline, glutamate, and alanine (PEA) at these positions whereas the vaccine strain M proteins have serine, lysine, and threonine (SKT). Extending our search to the totality of published SSPE sequences we were unable to find a single case where the M protein did not contain the PEA motif. However, in making a similar search of vaccine M proteins we found that they all have the SKT or PKT motif.

The present-day attenuated MeV vaccine strains were produced by passaging the original wt Edmonston strain and its derivatives on various nonhost animal cell lines [31]. Unfortunately, the original wt Edmonston strain is no longer available so that we can only speculate that the triresidue motif in its M protein was PEA. However, it can be concluded that this motif was at least PET as this is the nature of the motif in the minimally passaged “wt Edmonston.” It is interesting that modification of the triresidue motif appears to coincide with attenuation. Although circumstantial, this could suggest that replacement of the PEA or PET motif with SKT is involved, at least in part, in loss of virulence.

### 3.2. The B3 Genotype Lys-1 MeV Strain Has a Lowered Capacity for Virus Production

Interestingly, while all SSPE cases appear to be caused by wt MeV with the PEA motif in their M proteins, not all wt MeVs are PEA. Of the 23 wt MeV genotypes only 10 have had their M genes sequenced (Table 1). All have the PEA motif except the B3 genotype, which has PET. That the B3 genotype has the motif PET was of great interest to us for two reasons: (i) B3 is the prevalent genotype in Sub-Saharan Africa [21] and (ii) it has been observed [22] that, for unknown reasons, few cases of SSPE have been notified in this vast region where MeV is hyperendemic.

**Figure 2:**
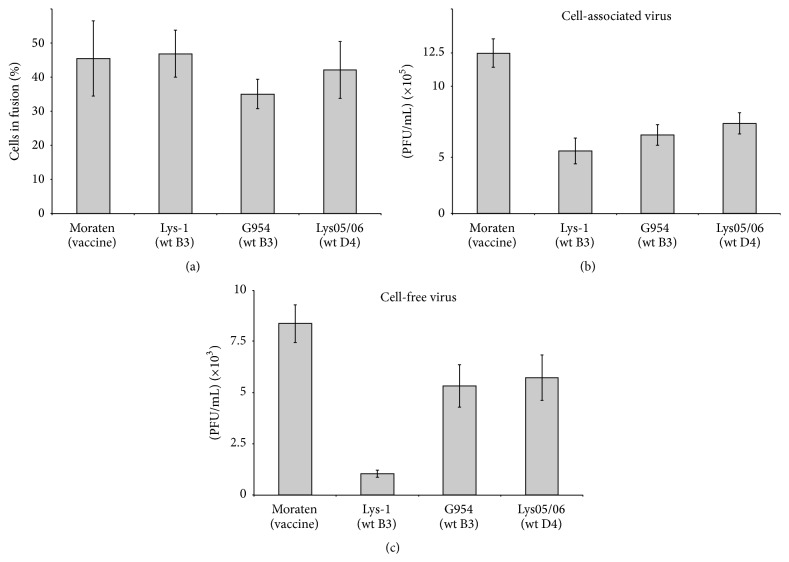
(a) Comparison of the cell-cell fusion capacity of Moraten (genotype A), Lys-1 (genotype B3), G954 (genotype B3), and Lys05/06 (genotype D4) strains. CHO-SLAM cells were infected with the different strains and the fusion levels analysed 30–36 h after infection. The histogram data represent the mean percentages ± standard deviations for three experiments. (b) Cell-associated virus production of the Moraten (genotype A), Lys-1 (genotype B3), G954 (genotype B3), and Lys05/06 (genotype D4) strains. CHO-SLAM cells infected with the different viruses were analysed for the production of cell-associated viral particles 48 h after infection. The histogram data represent the mean percentages ± standard deviations for three experiments. (c) Cell-free virus production of the Moraten (genotype A), Lys-1 (genotype B3), G954 (genotype B3), and Lys05/06 (genotype D4) strains. CHO-SLAM cells infected with the different viruses were analysed for the production of cell-free viral particles 48 h after infection. The histogram data represent the mean percentages ± standard deviations for three experiments.

Hypothesizing that the PET motif could potentially reduce the capability of the B3 genotype to spread within the human body, we compared two B3 genotype viruses (Lys-1 and G954) with a PEA motif-containing D4 genotype virus (Lys05/06) and the vaccine strain Moraten (SKT), for their cell-cell fusion and virus production capacities. As far as cell-cell fusion was concerned, we found little difference between the four viruses (Figure 2(a)). Although the fusion capacity of one of the PET motif-containing B3 strains (G954) was reduced by 17% in comparison with the PEA motif-containing D4 genotype virus Lys05/06 (Figure 2(a)), this was not statistically significant. For production of cell-associated virus, the three wt strains produced less than the vaccine Moraten strain (Figure 2(b)) and, comparing the B3 strains with the D4 strain, G954 had a 13% less production than Lys05/06 but again this reduction is not statistically important. However, for Lys-1 B3 the reduction in cell-associated virus compared to Lys05/06 was 32% (*P* < 0.025). Moreover, although there was only a slight (7%) reduction for G954, there was a significant reduction (82%; *P* < 0.001) in cell-free virus production for the Lys-1 B3 strain compared to the Lys05/06 D4 strain (Figure 2(c)).

Taken together, these results suggest that the Lys-1 B3 strain has a less productive phenotype than the Lys05/06 D4 strain but this is not the case for the G954 B3 strain. As both B3 strains contain the PET motif in their M proteins, this could thus suggest that the M protein PET motif has no influence on the phenotype of wt MeV strains in terms of virus production. However, upon sequencing the M gene of G954 we observed that residue 86, just three residues upstream of E89, was arginine (R) rather than lysine (K). This change both increases the positive charge of residue 86 [32] and introduces the possibility of cation-*π* interactions with aromatic residues [33] that could potentially play a compensatory role if, as has been previously suggested [34], the K89E mutation abrogates an electrostatic interaction between the M protein and the cytoplasmic tails of the glycoproteins which favors virus assembly.

In effect, our results show that the Moraten strain, which has the M protein motif SKT, exhibits much higher virus production levels than the PET or PEA motif-containing wt strains (Figures 2(b) and 2(c)). But is this difference related to the PEA/PET motifs? Evidently, to relate virus assembly differences between vaccine and wt MeV strains to the identity of a triresidue motif in the M protein, when variation also exists in other MeV proteins, in particular the H is pure speculation. We therefore undertook the construction of MeV recombinants to investigate the potential role, if any, that the M protein motif PEA plays in wt MeV production.

### 3.3. Production of Recombinant Moraten Viruses Differing according to the Nature of the Triresidue Motif in Their M Proteins

**Figure 3:**
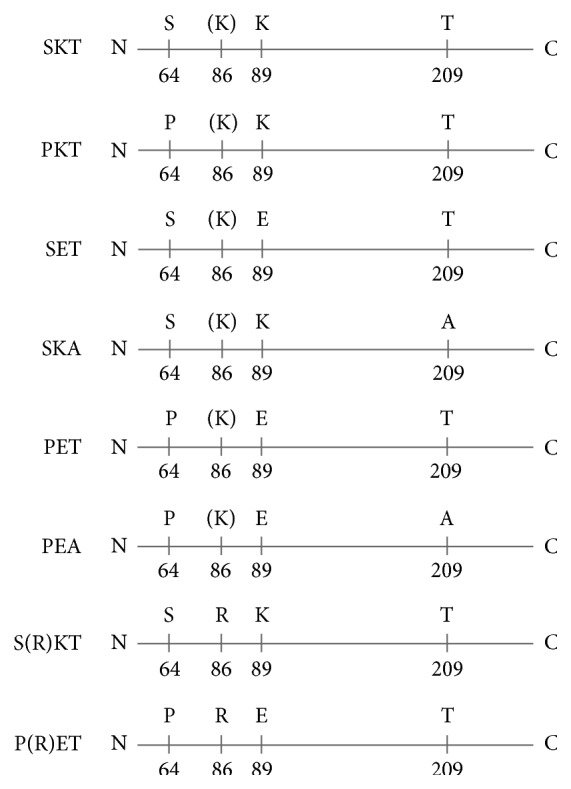
Nature of the M protein motif present in the different Moraten recombinants.

Recombinant Moraten viruses were built and rescued in which the triresidue SKT motifs present in the M protein of this vaccine strain were systematically replaced by elements from the wt motif PEA (Figure 3). These recombinant viruses are named according to the amino acids occupying the triresidue motif (aa 64, 89, and 209) of the M protein: SKT, PKT, SET, SKA, PET, and PEA. Two additional recombinants were made to test the effect of the K86R mutation: S(R)KT and P(R)ET (Figure 3). The phenotypes of these different recombinants were investigated with regard to their cell-cell fusion and virus assembly capacities. We used the Moraten reverse genetics system to do this study as a vaccine strain affords the possibility to test both the fusion and viral assembly capacities of recombinants in terms of differential receptor usage.

### 3.4. Substitution of the SKT Motif of the Moraten M Protein with Elements of the wt PEA Motif Results in an Increase in Cell-Cell Fusion

**Figure 4:**
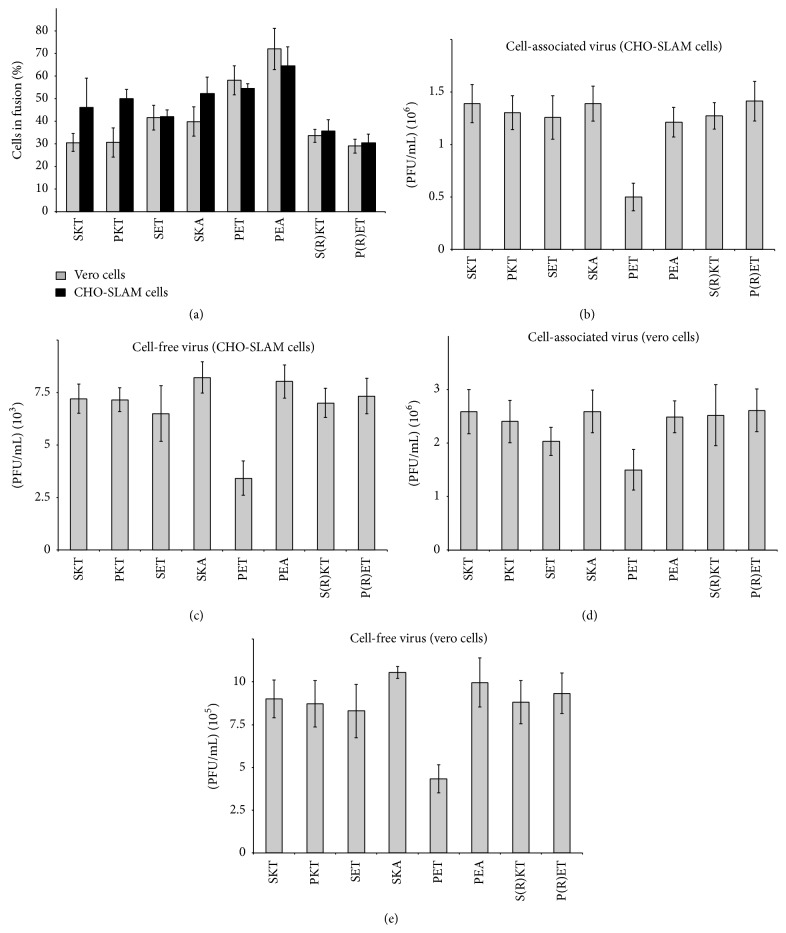
Assay of Moraten strain recombinants cell-cell fusion and virus assembly capacities. Vero cells and CHO-SLAM cells infected with Moraten recombinants were analysed 30 h and 48 h after infection for their cell-cell fusion and virus assembly capacities, respectively. Histogram data represent the mean percentages ± standard deviations for three experiments. (a) CD46- and SLAM-dependent fusion; ((b) and (c)) SLAM-dependent production of cell-associated and cell-free virus, respectively; ((d) and (e)) CD46-dependent production of cell-associated and cell-free virus, respectively.

The cell-cell fusion capacity of the different recombinants which have various permutations of the SKT and PEA motifs in their M proteins was compared using both CD46-expressing cells (vero) and SLAM-expressing cells (CHO-SLAM). Surprisingly, the results suggest that the identity of three particular residues in the M protein (at positions 64, 89, and 209) can have an effect on MeV cell-cell fusion levels. The results shown in Figure 4(a), comparing the levels of cell-cell fusion obtained for the various recombinants, suggest that whenever an element of the (vaccine) SKT motif is substituted by an element from the (wt) PEA motif, individually or in combination, there is an increase in cell-cell fusion even if we found that this is only statistically important for PET (*P* < 0.005) and PEA (*P* < 0.001). Interestingly, the highest values for both CD46-dependent cell-cell fusion and SLAM-dependent cell-cell fusion were obtained with the MeV recombinant containing the PEA motif in its M protein. This was particularly true for CD46-dependent fusion; the amount of cell-cell fusion generated by the PEA mutant was more than twice that generated by the SKT mutant (Figure 4(a)).

As a looser interaction between the M protein and the H and F proteins has been proposed to increase cell-cell fusion rates [2, 3], a possible explanation for our results is that these PEA-based substitutions in the Moraten M protein have loosened its interaction with the glycoprotein cytoplasmic tails. The substitution S64P does not appear to cause any increase in cell-cell fusion (comparing SKT with PKT) but the K89E substitution when allied with the S64P substitution (PET and PEA) increases both CD46- and SLAM-dependent fusion substantially.

### 3.5. A PET Motif in the Moraten M Protein Results in a >40% Reduction in Both Cell-Associated and Cell-Free Virus Production irrespective of Receptor Usage

We next examined the capacity of each recombinant to produce cell-associated and cell-free virus. As a tight interaction between the M protein and the glycoproteins has been reported to favor virus assembly [2, 3], the use of this assay should indicate the state of this interaction for each recombinant. The results obtained for CD46- and SLAM-dependent cell-associated virus production 2 days after infection (Figures 4(b) and 4(d)) suggest that PEA motif substitutions, with one exception, only have a slight negative effect on Moraten virus assembly. An exception however is the PET recombinant whose cell-associated virus production was reduced by more than 40% (Figures 4(b) and 4(d)). Very similar results were obtained for CD46- and SLAM-dependent cell-free virus production (Figures 4(c) and 4(e)).

The results obtained with the PET recombinant are in perfect accordance with our previous observation that Lys-1, a B3 genotype virus, gives very little cell-free virus (Figure 2(c)). This strongly suggests that the PET M protein motif in B3 genotype wt MeV viruses has a negative effect on virus production, possibly via modulation of assembly.

We also tested the P(R)ET recombinant, which differs from the PET recombinant only in having the K86R substitution, to determine whether this change is responsible for the higher level of virus production observed with the G954 B3 strain compared to the Lys-1 B3 strain.

### 3.6. Adding the Mutation K86R to the PET Recombinant Increases Virus Production but Lowers Fusion

We found that the P(R)ET recombinant indeed has compared with the PET mutant, a higher capacity (*P* < 0.001) for cell-free and cell-associated virus production with both SLAM-expressing cells and CD46-expressing cells (Figures 4(b), 4(c), 4(d), and 4(e)) and a lower fusion capacity (both SLAM-dependent and CD46-dependent) (Figure 4(a); *P* < 0.025). In fact, the P(R)ET recombinant has fusion and production properties identical to that of the parental S(K)KT Moraten virus. On the other hand, adding the K86R mutation to the SKT recombinant, S(R)KT, had little effect on virus production or cell-cell fusion (Figures 4(a), 4(b), 4(c), 4(d), and 4(e)). If the interaction between the Moraten M protein and the glycoprotein's cytoplasmic tails is indeed loosened by the K89E substitution, it is tempting to speculate that the K86R mutation restores the level of basic charge required for this interaction and that this tighter interaction is reflected in increased virus production and lower cell-cell fusion.

### 3.7. Confocal Microscopy Studies Show That MeV-M Proteins Associate with the H and F Glycoproteins irrespective of the Nature of the Triresidue Motif They Possess

**Figure 5:**
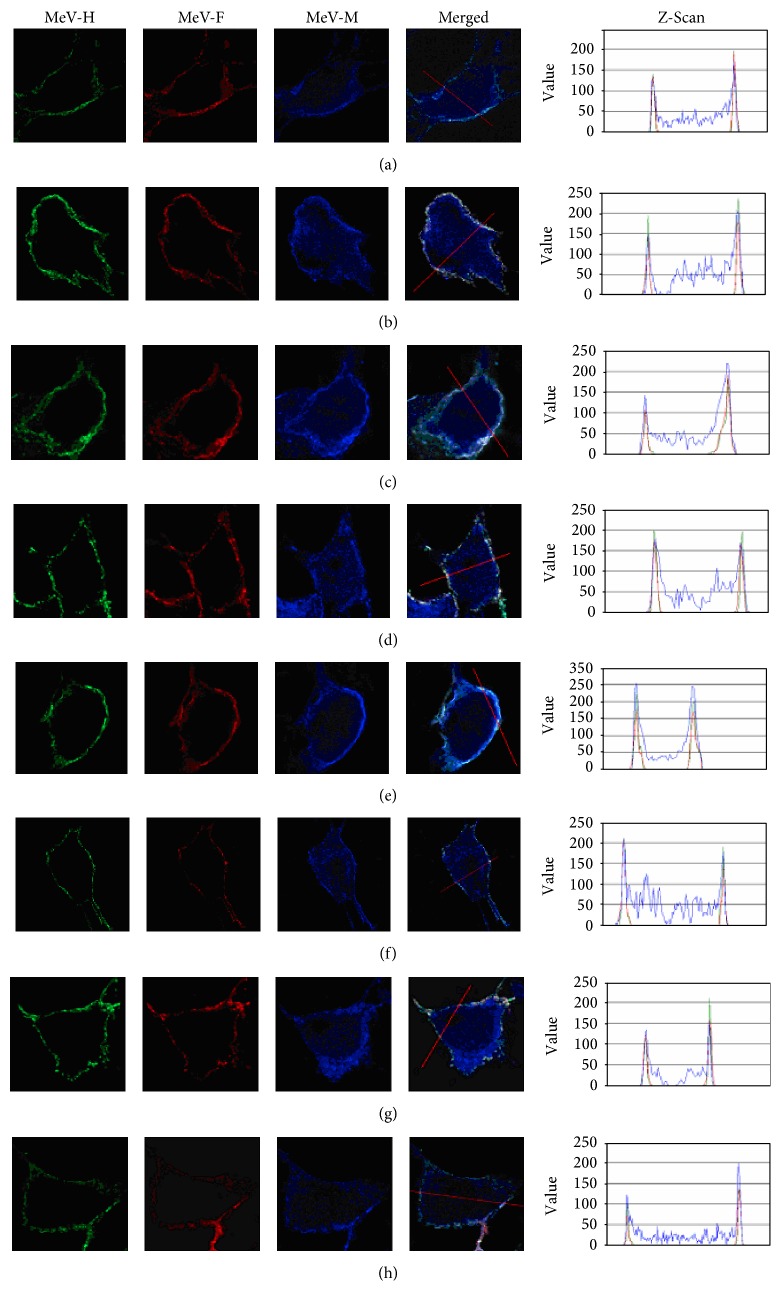
Confocal study of localization of MeV proteins, H, F, and M, of Moraten recombinant viruses. Vero-SLAM cells infected with these recombinant viruses were stained with anti-H labeled with Alexa Fluor 488, anti-F mAb Y503 with Alexa Fluor 555, and anti-M mAb8910 with Alexa Fluor 647. (a) SKT; (b) PKT; (c) SET; (d) SKA; (e) PET; (f) PEA; (g) S(R)KT; (h) P(R)ET.

To investigate whether substituting the Moraten SKT motif with elements of the wt PEA motif had an effect on the colocalization of the M protein with the H and F proteins, we made confocal microscopy studies. The colocalization of the Moraten M, H, and F proteins was not found to be affected by any of the PEA substitutions (Figure 5). This suggests that even if particular PEA substitutions have the effect to lessen or increase the interaction between the M protein and the cytoplasmic tails of the glycoproteins, these effects are probably subtle as they are not accompanied by topological displacement of the participating proteins.

## 4. Discussion

The reason why SSPE is caused exclusively by wt MeV and never by vaccine strains is not known. However, our results suggest that the capacity of wt MeV strains to cause SSPE results from their elevated capacity to spread and that this is due, at least in part, to a triresidue motif, PEA, in their M proteins. We thus propose the PEA motif as a molecular marker of wt MeV that risk causing SSPE.

Indeed, all SSPE cases reported in the literature have the PEA motif in their M proteins and we show that replacing the SKT motif in the Moraten vaccine M protein with the wt PEA motif increases fusion whilst maintaining virus production capacity. Moreover, changing this motif to PET via the single mutation A209T results in a significant reduction in virus production. Importantly, lowered virus production could hamper efficient viral spread in the CNS. Such attenuation could lessen the risk of B3 genotype viruses, which carry the PET motif rather than the PEA motif, to cause SSPE. Moreover, the general attenuated phenotype of vaccine strains would thus appear to preclude them from causing SSPE.

Importantly, our results suggest that the triresidue motif SKT (or PKT) in their M proteins could contribute to the attenuation of vaccine strains over and above their incapacity to act against the host innate immune response. But are vaccine strains with SKT more attenuated than those with PKT? Some MeV vaccine strains (Moraten, Schwarz, Rubeovax, and CAM70) have the SKT motif in their M proteins, whereas others (AIK-C, Zagreb, Leningrad16, Shanghai191, and Changchun47) have PKT. The Moraten, Schwarz, Rubeovax, AIK-C, and Zagreb vaccine strains have been adapted from the Edmonston-Enders strain, but with different passage histories [31]. As it has been shown that the AIK-C and Zagreb vaccine strains are more virulent than Moraten [35], it is tempting to speculate that this is due to the presence of a proline residue at position 64 in their M proteins rather than serine. It would be interesting to determine whether the introduction of the P64S mutation into the M proteins of these vaccine strains would lessen their virulence and thereby increase their safety.

Although Nigeria has had the highest rates for measles morbidity and mortality in the world [36], it was not until 1999 that field isolates of measles in this country, the most populous nation in Africa, were studied [37] and the B3 genotype was found to be prevalent. The B3 genotype is now the predominant genotype in Sub-Saharan Africa [21]. However, despite MeV's hyperendemicity, few SSPE cases have been reported in this vast region [22]. Moreover, despite an extended search of the literature and data banks, we have been unable to find a single SSPE case involving a B3 genotype wt virus [38]. Our results show that the M protein motif PET is associated with the reduced virus production of the B3 genotype virus Lys-1 possibly because of less efficient assembly. However, the PRET recombinant reveals that the additional mutation K86R is responsible for the normal levels of viral production exhibited by the other B3 genotype virus, G954. Although the G954 virus and its equivalent PRET recombinant exhibit lowered cell-cell fusion compared to the D4 genotype virus Lys05/06 and the PEA recombinant, we predict that if ever a B3 genotype virus case of SSPE appears, it will likely possess the K86R mutation in addition to PET. The chance of this occurring however is probably slight as G954 is the only B3 genotype virus (out of four) that has been shown, as yet, to possess the K86R mutation in the M protein.

Loosening the interaction between the M protein and the cytoplasmic tail of the H and F glycoproteins results in increased cell-cell fusion and less virus assembly; tightening this interaction leads to lower fusion and increased virus assembly [2, 3]. Moreover, in a study of the adaptation of wt MeV to vero cells, the introduction of an M gene coming from a vaccine strain (and thus the P64S, E89K, and A209T changes) into a wt MeV recombinant allowed growth in these CD46+/SLAM− cells, albeit with a low entry efficiency and no cell-cell fusion [39]. Observing that the essential difference between the vaccine M and the wt M was the identity at residues 64, 89, and 209, these authors then made wt MeV recombinants with the P64S, E89K, and A209T changes present both individually and in combination. They found that only the P64S and E89K changes allowed the wt MeV to grow well in vero cells; A209T had no effect. This is supported by PCR studies that have shown that when wt MeV adapts to vero cells, mutations can appear at P64 [40] and E89 [41], but there have been no reports of A209 being mutated.

In the following study [39], the Yanagi group (who discounted A209T) present results suggesting that the mutations P64S and E89K substitutions allow a strong interaction of the M protein with the cytoplasmic tail of the H protein and thereby an enhancement of virus assembly at the expense of cell-cell fusion. This fit well with our results, in that replacement of the SKT motif with PEA motif elements has the effect of increasing fusion although we do not see large decreases in virus production, except of course for the PET mutant. Using confocal microscopy, we did not obtain evidence for any change in the colocalization of H, F, and M proteins at the plasma membrane for any M mutant. In contrast, the Yanagi group study [39] found that some delocalization occurs but their results appear to reflect the topology of the three proteins on intracellular membranes rather than at the plasma membrane.

It would appear that adaptation of wt MeV to vero cells and SSPE pathogenesis have much in common. It has been known for over 25 years that wt MeV strains can adapt to vero cells (CD46+ but SLAM−) and thereby become attenuated. The virus enters by as yet unknown mechanism and replicates efficiently but, at least at first, there is no sign of cell-cell fusion despite multiple “blind” passages. Such persisting viruses have been shown to have mutations in the M protein, at either residue P64 [40] or residue E89 [41]. However, in some cases cell-cell fusion suddenly appears. This is triggered by mutations in the H protein such as N481Y [42] that allows the virus to use CD46 as receptor [28]. Presumably, the N481Y mutation changes the 3D conformation of the H protein so as to allow this change in receptor usage. We speculate that the PEA motif has its effect by a similar mechanism. Previously we showed that mutations in the M protein can affect the conformation of the F and H glycoproteins and thus facilitate resistance to neutralizing anti-MV sera [19]. Presumably, the PEA motif in the M proteins of wt MV strains induces a particular 3D conformation in the glycoproteins that allows an elevated immune escape and hence an increased potential for persistence.

In the case of SSPE, the virus enters brain cells such as neurons, again by an unknown mechanism, where it persists, accumulating mutations, mainly in the M protein but also in the H and F glycoproteins. After a long persistence usually lasting years, the symptoms of SSPE suddenly appear. However, perhaps not in all cases, it has been found, via autopsy examinations, that MeV “commonly” persists in the brains of healthy individuals [43]. We hypothesize that the “trigger” for this event is mutations that break the interaction between the M protein and the glycoprotein tails leading to accelerated spread of the virus in the brain and a resultant destructive inflammatory response. Such mutations, truncation of the M protein and/or F protein cytoplasmic tail, are found in the majority, if not all, SSPE cases where the pertinent sequences are available [38]. A possible means of MeV entry not involving fusion is macropinocytosis. Evidence is accumulating that MeV can enter cells by this endocytic mechanism [44, 45] but intriguingly, both for adaptation to vero cells and entry into neurons, the only known MeV receptor available is CD46.

Considering that the triresidue MeV-M protein motif PEA seems to be a molecular marker for SSPE, it would appear to be a priority to sequence the M proteins from the B1, B2, C1, D1, D2, D9, D10, E, F, G1, G2, H2, and d11 genotypes of wt MeV, to determine the nature of their triresidue motif and to make epidemiological correlations with the frequency of SSPE cases according to the circulating genotype. However, the underlying molecular mechanisms that allow wt MeV viruses with the PEA M molecular marker to slowly invade the CNS and give rise to SSPE remain unknown.

## 5. Conclusions

This study seeks to discover why SSPE is always caused by wt MeV, never by vaccine MeV. Our results suggest that SSPE can only be caused by MeV strains that contain a particular triresidue structural motif (PEA) in the primary sequence of their M proteins. Only wt MeV-M proteins contain the PEA motif. Results obtained from the construction of MeV recombinants suggest that the absence of this motif, in all MeV vaccine strains but also the wt B3 genotype, lowers the capacity of MeV to spread. Hence, we propose the MeV-M protein PEA motif as a molecular marker for MeV strains that risk causing SSPE.

## Figures and Tables

**Table 1:** Nature of the triresidue motif found in the gene encoding the matrix protein in the different wt MeV genotypes.

Genotype	M protein triresidue motif
**A**	SKT or PKT
B1	?
B2	?
**B3**	PET
C1	?
**C2**	PEA
D1	?
D2	?
**D3**	PEA
**D4**	PEA
**D5**	PEA
**D6**	PEA
**D7**	PEA
**D8**	PEA
D9	?
D10	?
E	?
F	?
G1	?
G2	?
**G3**	PEA
**H1**	PEA
H2	?
*d11*	?
